# Framingham cardiovascular disease risk scores and incident frailty: the English longitudinal study of ageing

**DOI:** 10.1007/s11357-014-9692-6

**Published:** 2014-08-02

**Authors:** Catharine R. Gale, Cyrus Cooper, Avan Aihie Sayer

**Affiliations:** 1 MRC Lifecourse Epidemiology Unit, University of Southampton, Southampton, SO16 6YD UK; 2 Centre for Cognitive Ageing and Cognitive Epidemiology, Department of Psychology, University of Edinburgh, Edinburgh, UK

**Keywords:** Frailty, Cardiovascular risk, Cohort, Longitudinal study

## Abstract

Cross-sectional studies show that frailty is common in older people with cardiovascular disease. Whether older people at higher risk of developing cardiovascular disease are more likely to become frail is unclear. We used multinomial logistic regression to examine the prospective relation between Framingham cardiovascular disease risk scores and incidence of physical frailty or pre-frailty, defined according to the Fried criteria, in 1,726 men and women aged 60 to over 90 years from the English Longitudinal Study of Ageing who had no history of cardiovascular disease at baseline. Men and women with higher Framingham cardiovascular risk scores were more likely to become frail over the 4-year follow-up period. For a standard deviation higher score at baseline, the relative risk ratio (95 % confidence interval) for incident frailty, adjusted for sex and baseline frailty status, was 2.76 (2.18, 3.49). There was a significant association between Framingham cardiovascular risk score and risk of pre-frailty: 1.69 (1.46, 1.95). After further adjustment for other potential confounding factors, the relative risk ratios for frailty and pre-frailty were 2.15 (1.68, 2.75) and 1.50 (1.29, 1.74), respectively. The associations were unchanged after excluding incident cases of cardiovascular disease. Separate adjustment for each component of the risk score suggested that no single component was driving the associations between cardiovascular risk score and incident pre-frailty or frailty. Framingham cardiovascular risk scores may be useful for predicting the development of physical frailty in older people. We now need to understand the biological mechanisms whereby cardiovascular risk increases the risk of frailty.

## Introduction

Frailty is a clinical syndrome observed in older people whose core feature is an increased vulnerability to stressors due to impairments in multiple, inter-related systems, decreased physiological reserves and a decline in the ability to maintain homeostasis (Bergman et al. 2007; Clegg et al. 2013). It is common (Gale et al. 2014a) and has numerous adverse consequences, including disability, falls, morbidity, hospitalization, institutionalization and death. Its causes are complex and likely to involve both biological and psychosocial mechanisms (Rockwood et al. 1994; Walston et al. 2006).

Cross-sectional surveys show that frailty and chronic disease frequently co-exist (Wong et al. 2010). One such disease whose relationship with frailty has been the focus of many studies is cardiovascular disease (CVD) (Afilalo et al. 2014; von Haehling et al. 2013). A systematic review of studies based on over 54,000 community-dwelling older people found that the odds ratios for prevalent frailty associated with CVD ranged from 2.7 to 4.1 (Afilalo et al. 2009) and that over a mean follow-up period of 6 years, the presence of CVD increased the risk of incident frailty by 50 %. These findings suggest that CVD risk may be predictive of future frailty and raise the possibility that multivariable risk factor algorithms for assessing CVD risk in primary care, such as that developed as part of the Framingham Heart Study (D’Agostino et al. 2008), might provide a method for identifying individuals currently free of CVD who are at increased risk of becoming frail. Longitudinal studies in the Whitehall II cohort have shown that in middle-aged people without a history of stroke or heart disease, higher Framingham CVD risk scores are associated with poorer performance on objective tests of physical function (Elbaz et al. 2014) and with increased later risk of frailty (Bouillon et al. 2013), as defined by the Fried physical frailty phenotype (Fried et al. 2001). Whether such scores can predict future risk of frailty defined by the Fried phenotype in older people is unknown.

The English Longitudinal Study of Ageing (ELSA) is a large population-based sample of older men and women. We used these data to investigate the prospective relation between Framingham CVD risk scores and risk of incident frailty in men and women aged 60 to over 90 years (Marmot et al. 2013).

## Methods

### Participants

The data for this study come from the ELSA. The sample for ELSA was based on people aged ≥50 years who had participated in the Health Survey for England in 1998, 1999 or 2001 (Steptoe et al. 2013). It was drawn by postcode sector, stratified by health authority and proportion of households in non-manual socioeconomic groups. A total of 11,392 people participated in wave 1 in 2002–2003. At wave 2 in 2004–2005 and at wave 4 in 2008–2009, core sample members (those in the household aged ≥50 years who had taken part in wave 1) who had completed the main interview were invited to have a visit from a nurse that included measurements of physical function and blood pressure, anthropometry and blood sampling. Ethical approval was obtained from the Multicentre Research and Ethics Committee. Participants gave written informed consent.

### Measures

#### Frailty

Maximum handgrip strength was measured three times on each side using a dynamometer; the best of these measurements was used for analysis. Height and weight were measured with a portable stadiometer and electronic scales, respectively. Body mass index (BMI) was calculated as weight (in kilograms)/height (in metres)^2^. Gait speed was assessed in participants aged 60 and over by measuring the time taken to walk a distance of 8 ft at usual pace; the timed walk was repeated, and the mean of the two measurements was calculated. Participants responded to three questions about the frequency with which they did vigorous, moderate or mild exercise. We ranked the combinations of responses to these questions according to the amount and intensity of exercise involved to provide an estimate of usual physical activity. Symptoms of depression were assessed using the eight-item version of the Centre for Epidemiologic Studies Depression Scale (CES-D) (Steffick and The HRS working group 2000). We used these data, together with information on participants’ weight at the initial survey, to derive an indicator of physical frailty at wave 2 (baseline) and at wave 4 in men and women aged ≥60 years using the Fried criteria (Fried et al. 2001). These criteria define physical frailty as the presence of three or more of the following: unintentional weight loss, weakness, self-reported exhaustion, slow walking speed and low physical activity. We operationalized these criteria using definitions very similar to those used in the original phenotype of frailty studies (Bandeen-Roche et al. 2006; Fried et al. 2001): Weight loss was defined as *either* loss of ≥10 % of body weight since the initial survey (for frailty at wave 2) or since wave 2 (for frailty at wave 4) *or* current BMI <18.5 kg/m^2^; weakness was defined as maximum grip strength in the lowest 20 % of the distribution, after taking sex and BMI into account; exhaustion was considered present if the participant gave a positive response to either of the CES-D questions ‘Felt that everything I did was an effort in the last week’ or ‘Could not get going in the last week’; slow walking speed was defined as a walking speed in the lowest 20 % of the distribution, after taking account of sex and height; participants who did not perform the timed walk because they were unable to walk alone or had health restrictions were also categorised as having slow walking speed;and low physical activity was defined as physical activity in the lowest sex-specific 20 % of the distribution.

#### Framingham cardiovascular risk score at baseline

The Framingham general cardiovascular risk score was developed for use in primary care to assess general cardiovascular risk and risk of individual events (coronary, cerebrovascular, peripheral arterial disease and heart failure) (D’Agostino et al. 2008). Its sex-specific algorithm is based on age, HDL and total cholesterol, systolic blood pressure, treatment for hypertension, smoking and diabetes.

Data on the risk score components was taken from the wave 2 examination. Blood samples were taken from all participants except those who were not willing to give written consent, those with clotting or bleeding disorders and those taking anti-coagulant drugs. Samples were assayed for total and HDL cholesterol, haemoglobin A1C and in addition for fibrinogen and C-reactive protein at the Royal Victoria Infirmary, Newcastle-upon-Tyne, UK. Systolic blood pressure was measured three times using an Omron blood pressure monitor with the participant seated. We used the mean of these three values in our analysis. Participants were asked whether they were currently taking any medication for high blood pressure and about their current smoking status. Prevalent diabetes mellitus was defined based on reported doctor-diagnosed diabetes and/or use of diabetes medication, or a haemoglobin A1C level ≥6.5 % (The International Expert Committee 2009).

### Covariates at baseline

We chose cognitive function, household wealth and BMI as covariates because they could potentially confound any association between Framingham CVD risk score and later risk of frailty. Poorer cognition, lower socioeconomic status, as measured by household wealth, and higher BMI are independent predictors of frailty in both men and women in this cohort (Gale et al. 2014b), and have been shown to correlate with higher Framingham CVD risk scores (Elbaz et al. 2014; Kaffashian et al. 2013). Participants took tests of cognitive function as follows: Verbal memory (immediate and delayed recall of ten aurally presented nouns) was assessed using word lists developed for the US Health and Retirement Study (Ofstedal et al. 2005); prospective memory (remembering to do a specific task) and attention (letter cancellation task) were assessed using tests previously used in the MRC Cognitive Function and Ageing Study (Brayne et al. 1998); and executive function was assessed using a test of verbal (semantic) fluency (naming as many animals as possible in 60 s) taken from the CAMCOG-R (Roth et al. 1999). A total cognitive function score was calculated by summing scores on these tests. Socioeconomic status was indexed by total household wealth, including savings and investments, value of any property or business assets, net of debt, excluding pension assets. Household wealth has been identified as the most accurate indicator of long-term socioeconomic circumstances in ELSA (Banks et al. 2003).

In a previous study of this cohort, higher concentrations of the inflammatory markers fibrinogen and C-reactive protein were significant predictors of incident frailty in women, though not in men (Gale et al. 2013). As higher levels of these biomarkers correlate with higher Framingham CVD risk scores (Noh et al. 2013; Park et al. 2010), they might potentially confound any link between these risk scores and frailty, at least in women. We therefore carried out a subset analysis in which we examined whether additional adjustment for concentrations of fibrinogen and C-reactive protein altered the association between Framingham CVD scores and risk of frailty.

### Analytical sample

Of the 5,918 study members aged ≥60 years who were interviewed at wave 2 in 2004–2005, 5,377 agreed to be visited by a nurse (91 %). Of these, 3,454 were interviewed and visited by a nurse at wave 4 in 2008–2009. We excluded prevalent cases of CVD at the wave 2 baseline (reported doctor diagnoses of angina, heart attack, congestive heart failure or stroke). The present analysis is based on 1,726 people who were free of CVD at baseline and had complete data on Framingham cardiovascular risk score and baseline covariates at wave 2 and frailty at wave 4.

### Statistical analysis

We used ANOVA and chi-square test to examine differences in baseline characteristics according to the presence of pre-frailty or frailty at follow-up. We used multinomial logistic regression to examine the relation between a standard deviation increase in Framingham CVD risk score at baseline and risk of incident pre-frailty or frailty. We adjusted for sex and frailty status at baseline and then in addition for cognitive function, household wealth and BMI. In a subset of women only, we carried out an additional adjustment for the inflammatory biomarkers fibrinogen and C-reactive protein. In order to examine which components of the Framingham CVD risk score helped to explain its association with incident frailty, we examined the effect on the association of adjusting for each component (age, HDL and total cholesterol, systolic blood pressure, treatment for hypertension, smoking and diabetes) in turn.

All data were weighted to correct for sampling probabilities, non-response, and for differential sample loss between waves in order to make them more closely reflect the population from whom the ELSA sample was drawn. Detailed descriptions of these weights and their calculation can be found in the technical reports on the study available at www.ifs.org.uk/elsa.

## Results

Table 1 shows the baseline characteristics of the 1,726 men and women in the study according to whether they were not frail, pre-frail or frail at follow-up. The weighted percentage of participants who were pre-frail or frail at follow-up was 43 and 13 %, respectively. Mean Framingham CVD risk score at baseline rose with increasing degree of frailty at follow-up and differed significantly between those who were not frail, pre-frail or frail. As regards to the components of the Framingham CVD risk score, men and women who were older or who had diabetes at baseline tended to have a higher degree of frailty at follow-up. People who were pre-frail had higher systolic blood pressure at baseline than those who were not frail, but there were no differences in blood pressure between those who were pre-frail and those who were frail. Compared to those who were not frail at follow-up, those who were pre-frail were slightly more likely to have been a smoker at baseline, but this was of borderline significance, and there were no differences in baseline smoking status between those who were pre-frail and those who were frail at follow-up. Blood levels of total or HDL cholesterol and use of anti-hypertensive medication at baseline did not vary significantly by frailty status at follow-up. As regards to the covariates, cognitive function was poorer, level of household wealth lower and BMI higher at baseline in those with a greater degree of frailty at follow-up. There was no difference in sex distribution between those who were not frail and those who were pre-frail at follow-up, but there was a significantly higher proportion of women among those who were frail.

**Table 1:** Baseline characteristics of the study participants (*n* = 1,726) according to frailty status at follow-up

	Not frail (*n* = 862)	Pre-frail (*n* = 691)	Frail (*n* = 173)	P for difference between not frail and pre-frail	P for difference between pre-frail and frail
Framingham CVD risk score, mean (SD)	14.3 (2.59)	15.3 (2.48)	16.1 (2.35)	<0.0001	<0.0001
*Framingham CVD risk score components*					
Age (years), mean (SD)	67.1 (6.20)	71.6 (7.33)	76.2 (7.83)	<0.0001	0.005
HDL cholesterol (mg/dl), mean (SD)	61.0 (16.1)	60.1 (15.0)	60.7 (14.3)	0.261	0.267
Total cholesterol (mg/dl), mean (SD)	236.8 (50.2)	236.6 (46.2)	235.9 (47.1)	0.838	0.705
Current smoker, %	7.40	9.95	12.9	0.086	0.334
Systolic blood pressure (mm Hg), mean (SD)	135.9 (19.9)	139.5 (19.0)	141.2 (19.0)	0.001	0.465
On anti-hypertensive medication, %	13.7	16.0	15.5	0.238	0.882
Diabetes, %	5.99	7.16	16.6	0.001	0.004
*Covariates*					
Female, %	55.1	56.6	67.7	0.573	0.012
Cognitive function, mean (SD)	30.2 (5.74)	27.5 (5.52)	24.7 (4.96)	<0.0001	<0.0001
Lowest quintile of household wealth, %	9.09	18.5	29.4	<0.0001	<0.0001
BMI (kg/m^2^), mean (SD)	27.2 (4.24)	27.5 (4.39)	28.7 (5.24)	0.031	0.006
No of frailty criteria present, mean (SD)	0.29 (0.65)	0.79 (0.85)	1.65 (1.14)	<0.0001	<0.0001

Preliminary analyses showed that the relationship between Framingham CVD risk score and risk of incident pre-frailty or frailty did not differ between the sexes (*p* for interaction term >0.5). Multivariate multinomial logistic regression analyses of frailty risk were therefore carried out in men and women together, adjusting for sex.

Table 2 shows relative risks for incident pre-frailty and frailty for a standard deviation increase in Framingham CVD risk score. Results are shown adjusted first for sex and baseline frailty status, and then with further adjustment for the other potential confounding variables, household wealth, cognitive function and BMI. In models adjusting for sex and baseline frailty status, there was a significant positive association between Framingham CVD risk score at baseline and risk of incident frailty: For a standard deviation increase in the Framingham risk score, the relative risk ratio (RR) (95 % confidence interval) for incident frailty was 2.76 (2.18, 3.49). Further adjustment for cognitive function, household wealth and BMI at baseline only partially attenuated the association: RR (95 % CI) 2.15 (1.68, 2.75). There was a significant association between total score for Framingham CVD risk score at baseline and risk of incident pre-frailty: For a standard deviation increase in the Framingham risk score, the relative risk ratio (95 % confidence interval) for incident pre-frailty was 1.69 (1.46, 1.95). Further adjustment for cognitive function, household wealth and BMI at baseline had only minor attenuating effects: RR (95 % CI) 1.50 (1.29, 1.74).

**Table 2:** Relative risk ratios (95 % CI) for incident pre-frailty or frailty for a standard deviation increase in Framingham CVD risk score at baseline

	RR (95 % CI), adjusted for sex and baseline frailty status		RR (95 % CI), fully adjusted^1^	
	Pre-frailty	Frailty	Pre-frailty	Frailty
Framingham CVD risk score, per SD increase	1.69 (1.46, 1.95.)	2.76 (2.18, 3.49)	1.50 (1.29, 1.74)	2.15 (1.68, 2.75)

^1^Adjusted for sex, household wealth, cognitive function, BMI, and whether non-frail, pre-frail or frail at baseline

None of the participants in our sample had a reported history of CVD at baseline. To examine whether the associations between baseline Framingham CVD risk score and incident frailty or pre-frailty were concentrated among those who reported that they had been newly diagnosed with CVD during the 4-year follow-up period, we repeated our analysis after excluding this group (*n* = 68). Results were essentially unchanged.

In a subset analysis of the women in our sample (*n* = 982), we examined whether concentrations of the inflammatory markers fibrinogen and C-reactive protein—previously shown to be predictive of frailty in women only (Gale et al. 2013)—were confounders of the associations between baseline Framingham CVD risk score and incident frailty or pre-frailty. Adjustment for these biomarkers had little attenuating effect. For an SD increase in Framingham CVD score, the relative risk ratios for incident pre-frailty or frailty in women, adjusted for baseline frailty status, were 1.63 (1.34, 1.98) and 2.51 (1.87, 3.38), respectively. After further adjustment for baseline fibrinogen and C-reactive protein levels, these relative risk ratios were 1.61 (1.32, 1.96) and 2.34 (1.73, 3.18), respectively.

In order to investigate whether the associations observed between the Framingham CVD score at baseline and risk of incident frailty or pre-frailty were driven by particular components of the score (age, systolic blood pressure, use of anti-hypertensive medication, current smoking, HDL and total cholesterol and diabetes), we examined the effect on the sex and baseline frailty status-adjusted relative risks of further adjustment for each component in turn. The associations between CVD risk score and risk of pre-frailty and frailty were in almost all cases unattenuated by these adjustments (Table 3). The largest attenuating effects occurred when the associations between Framingham CVD risk score and risk of pre-frailty or frailty were adjusted for age, but they remained statistically significant: Relative risk ratios for pre-frailty or frailty changed from 1.69 (1.46, 1.95) and 2.76 (2.18, 3.49) to 1.24 (1.05, 1.46) and 1.71 (1.31, 2.24), respectively. Adjustment for diabetes, the only component of the Framingham risk score apart from age that was consistently and strongly linked with frailty status (see Table 1), had no attenuating effect on the associations between CVD risk score and risk of pre-frailty and frailty. To explore whether the associations might be better explained by a continuous measure of glucose metabolism, we adjusted for levels of haemoglobin A1C, but this too had no attenuating effect (data not shown). These results suggest that no specific component risk factor drives the associations between Framingham CVD risk score and risk of pre-frailty or frailty.

**Table 3:** Relative risk ratios (95 % CI) for incident pre-frailty or frailty for a standard deviation increase in Framingham CVD risk score at baseline, separately adjusted for each component of the score

Framingham CVD risk score, per SD increase	RR (95 % CI	
	Pre-frailty	Frailty
Adjusted for sex and baseline frailty status	1.69 (1.46, 1.95)	2.76 (2.18, 3.49)
Further adjusted for:		
Age	1.24 (1.05, 1.46)	1.71 (1.31, 2.24)
Total cholesterol	1.74 (1.50, 2.02)	2.89 (2.28, 3.67)
HDL cholesterol	1.68 (1.45, 1.84)	2.87 (2.25, 3.67)
Systolic blood pressure	1.63 (1.46, 1.95)	2.62 (2.07, 3.33)
Anti-hypertensive treatment	1.65 (1.45, 1.87)	2.76 (2.18, 3.49)
Smoking	1.69 (1.46, 1.95)	3.13 (2.43, 4.04)
Diabetes	1.80 (1.53, 2.11)	2.77 (2.12, 3.61)

## Discussion

In this prospective study of men and women aged 60 to over 90 years, none of whom had a history of CVD, higher Framingham CVD risk scores at baseline were associated with an increased risk of incident frailty and pre-frailty. Adjustment for potential confounding factors had only small attenuating effects on these associations, and they were little changed by the exclusion of those who developed CVD during follow-up.

To our knowledge, only one previous study has examined the relation between Framingham CVD risk scores and subsequent frailty. In the Whitehall II study, an SD increase in Framingham CVD score in people aged 45–69 years increased the odds of being frail 10 years later by 42 % (Bouillon et al. 2013). As in the present study, no single component of the CVD risk score appeared to be driving its association with future frailty. Direct comparison of effect sizes with those found in our study is difficult because we used multinomial rather than binary logistic regression so that we could examine whether Framingham CVD score was linked with risk of pre-frailty as well as frailty, but our findings suggest that the risk of frailty associated with raised Framingham CVD scores in older people is at least as high and possibly higher than that observed in middle-aged individuals. Furthermore, our results suggest that these CVD scores are also predictive of risk of pre-frailty—defined by Fried as the presence of just one or two of the five components of frailty (Fried et al. 2001). This has potential implications for prevention or amelioration. Whereas transitions to a state of greater frailty are more common than movement in the reverse direction, there is sufficient evidence of the latter to suggest that frailty is potentially reversible, at least in the less severe stage (Espinoza et al. 2012; Gill et al. 2006).

Several mechanisms could potentially underlie the association we observed between Framingham CVD risk scores in people with no known history of CVD and later risk of frailty. One possibility is that atherosclerosis affects blood flow to the nerves and muscles of the legs, exacerbating sarcopenia, a major component of frailty (Morley et al. 2002). There is some evidence that the prevalence of frailty is higher in older people with peripheral atherosclerosis as indicated by a high ankle-brachial index or other indicators of subclinical CVD such as carotid stenosis or left ventricular hypertrophy (Newman et al. 2001; Singh et al. 2012). In the Three Cities study of older people, mean walking speed decreased with increasing number of carotid plaques and increased intimal-medial thickening (Elbaz et al. 2005). Another related factor might be brain pathology. Brain autopsy in members of two US cohorts—the Religious Orders Study and the Rush Memory and Aging Project—followed for up to 14 years showed that the presence of subclinical CVD and Alzheimer pathologies was associated with more rapid progression of frailty, and in particular with more rapid decline in walking speed (Buchman et al. 2013). Although inflammation is a plausible underlying mechanism, given observations in several studies linking higher levels of inflammatory biomarkers with higher CVD risk scores (Noh et al. 2013; Park et al. 2010) and increased risk of incident frailty (Baylis et al. 2012; Puts et al. 2005), we found little evidence that it played a part in the associations we found. Another potential underlying mechanism is impaired glucose metabolism. There is evidence that muscle strength and physical function are poorer in older people with raised glucose levels, and this is not confined to those with diabetes (Sayer et al. 2005). Diabetes has been linked with increased risk of becoming frail (Lee et al. 2014), perhaps by accelerating age-related muscle loss (Kalyani et al. 2014). However, in this study, although the presence of diabetes at baseline was strongly associated with greater frailty at follow-up, neither it nor levels of a baseline marker of glucose metabolism—haemoglobin A1C—helped to explain the association between Framingham CVD risk scores and incident pre-frailty or frailty.

The strengths of our study include the large sample size, the fact that ELSA was designed to be representative of the older community-dwelling English population (Steptoe et al. 2013), and our use of a prospective study design in which we assessed incident cases of pre-frailty or frailty that occurred after the measurement of CVD risk factors. There are also some weaknesses. First, information on prevalent and incident CVD was based solely on reported doctor diagnoses of angina, heart attack, congestive heart failure or stroke. Second, not all the participants in the baseline survey gave a blood sample (77 % of those interviewed); non-responders to the blood sample tended to be older and in poorer health. However, in view of the fact that the associations we found between Framingham CVD risk score and risk of frailty were at least as strong as those reported in a previous study of middle-aged people (Bouillon et al. 2013), it seems unlikely that our findings would be very different if we had validated data on history of CVD and complete data on blood biomarkers for all those who were interviewed at baseline. Finally, our follow-up period was relatively short—around 4 years—which raises the possibility of reverse causation whereby the higher baseline Framingham CVD risk scores observed in people who were frail or pre-frail at follow-up might be a consequence of their prior frailty status. We can be fairly sure that this is not the case as the association we found was robust to adjustment for the number of components of frailty present at baseline. Furthermore, it is consistent with observations from other longitudinal studies with much longer follow-up times that higher levels of cardiovascular risk factors in midlife—whether assessed as overweight or obesity (Stenholm et al. 2014), physical activity (Savela et al. 2013) or a composite risk factor score for coronary artery disease (Strandberg et al. 2012)—increase the risk of frailty over 20 years later.

In this prospective study of men and women aged 60 to over 90 years, none of whom had a history of CVD; higher Framingham CVD risk scores were associated with an increased risk of incident frailty and pre-frailty. Use of these scores in clinical practice may help identify those older people who are at risk of becoming frail. We now need to understand the biological mechanisms whereby CVD risk increases the risk of frailty.
